# Foreword to the AfCA collection: celebrating work published by African researchers in IUCr journals

**DOI:** 10.1107/S2056989024008272

**Published:** 2024-09-30

**Authors:** Susan A. Bourne, Delia A. Haynes, Michele Zema

**Affiliations:** a Department of Chemistry, University of Cape Town, Rondebosch, South Africa; b Department of Chemistry & Polymer Science, Stellenbosch University, Matieland, South Africa; c International Union of Crystallography,Chester United Kingdom; d Department of Earth and Geoenvironmental Sciences University of Bari ‘Aldo Moro’ Bari Italy

**Keywords:** AfCA, crystallography, Africa, IUCr

## Abstract

This virtual collection is a celebration. It is a celebration of crystallography in Africa, and a celebration of the international crystallography community. It commemorates the founding of the African Crystallographic Association (AfCA), which was accepted as the newest Regional Associate of the International Union of Crystallography (IUCr) in 2023, the year of the 75th anniversary of the first IUCr Congress and General Assembly. The collection contains research articles authored by scientists across the African continent, as well as a selection of articles giving context to crystallography in Africa. These discuss history, collaboration between scientists and between associations, and various educational and outreach initiatives.

This virtual collection is a celebration. It is a celebration of crystallography in Africa, and a celebration of the international crystallography community. It commemorates the founding of the African Crystallographic Association (AfCA), which was accepted as the newest Regional Associate of the International Union of Crystallography (IUCr) in 2023, the year of the 75th anniversary of the first IUCr Congress and General Assembly. The collection contains research articles authored by scientists across the African continent, as well as a selection of articles giving context to crystallography in Africa. These discuss history, collaboration between scientists and between associations, and various educational and outreach initiatives.

Africa is a fascinating and diverse continent. It has the world’s youngest population, more than 50 countries, well over 1000 languages, and a wealth of natural resources. Despite this huge potential, Africa is the least wealthy continent per capita, and science remains underdeveloped on the continent as a whole. There are many complex reasons for this. For example, it is interesting to note that in 1947, when the IUCr was founded, only five African countries were not colonies. Recent years have shown great developments in science in Africa, often driven by African scientists who want to develop African solutions to African and global problems.

As all crystallographers know, crystallography is a key science, underpinning developments in a variety of diverse fields from biology to mineralogy and materials science. The potential of crystallography to make key contributions to addressing a variety of challenges is undisputed. There is therefore great value in developing crystallography as a science in Africa.

Through the support of crystallographers worldwide, particularly under the auspices of the IUCr and its Crystallography in Africa initiative (Lecomte, 2017 ▸), and of the European Crystallographic Association (ECA), the teaching and use of crystallography on the African continent is growing and developing at a rapidly increasing rate, which is shown by the articles in this collection. There are regular schools and workshops across the continent, and the IUCr–UNESCO OpenLabs (https://www.iucr.org/outreach/openlabs; Zema & Lecomte, 2015 ▸; Zema *et al.*, 2017 ▸) continue to be extremely successful. Through this initiative, crystallographic educational and research centres are being implemented at existing centres of competency. Building on the expertise gained through these actions, brand new developments are being established promoting regional research excellence and building capacity in multidisciplinary research based around a new well equipped X-ray laboratory. These new facilities become a hub for regional excellence. This is the case for the X-TechLab project at Sèmè City in Benin (https://www.xtechlab.co/; Agbahoungbata *et al.*, 2021 ▸), which is an outgrowth of the IUPAP–IUCr–ICTP Lightsources for Africa, the Americas, Asia, Middle East and Pacific (LAAAMP) initiative (https://laaamp.iucr.org; Scandolo *et al.*, 2017 ▸) in collaboration with the government of Benin.

Four new crystallographic associations have been inaugurated in Africa in recent years (Senegal, Gabon, Republic of Congo and Ivory Coast). There are also regular scientific meetings and conferences involving crystallography around Africa. The Pan African Conference on Crystallography (PCCr) series is now well established, with PCCr4 set to take place in Algeria in 2025. The COVID-19 pandemic actually had some positive outcomes in terms of the development of crystallography in Africa – the increased use of online tools enabled African crystallographers to meet and network much more easily, rapidly advancing the formalization of a crystallographic association. Thus, the African Crystallographic Association was officially launched in 2021. In 2023, at the General Assembly of the IUCr in Melbourne, AfCA was accepted as the fifth Regional Associate of the IUCr. This is a truly significant milestone for crystallography in Africa.

The history of the AfCA has involved a long road and a lot of hard work by many people around the globe. In his article ‘When a dream comes true: birth of the African Crystallographic Association (AfCA)’, Andreas Roodt (2024 ▸) tracks this history. He begins with the early days of crystallography in the North (Egypt, Tunisia, Algeria and Morocco) and South Africa, highlighting the role played by various individuals, and by the IUCr, particularly through the IUCr Crystallography in Africa initiative and the International Year of Crystallography in 2014.

As pointed out by Roodt, access to infrastructure is a significant challenge to many crystallographers in Africa. Not only is acquiring instrumentation difficult, but stable electricity and water supplies cannot always be taken for granted. In addition, research funding is limited or non-existent in many African countries. There are several approaches that African researchers follow to tackle these challenges. One of these is to foster collaborations with institutes that have the required facilities. The benefits of collaboration are elegantly illustrated in the article co-authored by researchers from the universities of Dakar in Senegal and Southampton in the UK (Orton *et al.*, 2023 ▸). This highly productive collaboration is an example of what can arise from networking between scientists in Africa and those from around the world, as emphasized in the article.

The importance of collaboration is also highlighted in Simon Billinge’s article about JUAMI, the Joint Undertaking for an African Materials Institute (Billinge, 2024 ▸). As described by Billinge, JUAMI is a ‘community-building exercise’, and an exciting example of how training and networking can lead to valuable and productive collaboration. The number of exciting projects that have developed form these schools is inspiring. Another recent approach to solving the infrastructure challenge is the concept of a ‘remote lab’. This has been driven by researchers at the University of Lorraine in Nancy, France, and is described in the article by Kenfack Tsobnang *et al.* (2024 ▸). This project provides intense crystallographic training to researchers at African institutes, who are then able to access a diffractometer remotely and control their own data collections. This approach has been successfully used for teaching at several workshops and OpenLabs. Most excitingly, one of the first crystal structures obtained using the remote lab concept has been published as part of this collection (Kenfack Tsobnang *et al.*, 2024 ▸).

The development of crystallography in Africa is part of a wider drive to develop science in Africa. This strong drive is exemplified by the African Light Source Foundation (AfLS). The progress of the AfLS is presented in the article by Connell *et al.* (2024 ▸) in this collection. The article discusses the significant advances made towards building an advanced light source on the African continent and highlights the importance of societies working together and the value of science diplomacy.

The first two objectives of the IUCr, as stated in the statutes, are ‘to promote international cooperation in crystallography’, and ‘to contribute to the advancement of crystallography in all its aspects.…’. It is clear from this virtual collection that the work of the IUCr in promoting crystallography in Africa is fulfilling these objectives. Despite many challenges, African crystallographers are producing excellent science. Crystallography in Africa has come of age, and this virtual collection reflects that.

## References

[bb1] Agbahoungbata, M. Y., Bonou, S. A. S., d’Almeida, T., Zema, M., Mtingwa, S. & Borna, C. (2021). *Acta Cryst.* A**77**, C606.

[bb2] Billinge, S. J. L. (2024). *Acta Cryst.* E**80**, 102–105. 10.1107/S2056989023010915 PMC10848968 38333138

[bb3] Connell, S. H., Dollman, K., Kamel, G., Khan, S. A., Mitchell, E., Mtingwa, S. K., Newton, M. C., Ngabonziza, P., Nji, E., Norris, L. & Zema, M. (2024). *J. Synchrotron Rad.* **31**, 1–9. 10.1107/S1600577523009682 PMC10833437 38142406

[bb4] Kenfack Tsobnang, P., Ziki, E., Siaka, S., Yoda, J., Kamal, S., Bouraima, A., Djifa Hounsi, A., Wenger, E., Bendeif, E.-E. & Lecomte, C. (2024). *Acta Cryst.* E**80**, 106–109. 10.1107/S2056989023011052 PMC10848970 38333131

[bb5] Lecomte, C. E. P. (2017). *Acta Cryst.* A**73**, C1394.

[bb6] Orton, J. B., Diouf, N., Gueye, R. S., Gaye, M., Thiam, I. E. & Coles, S. J. (2023). *Acta Cryst.* E**79**, 1109–1114. 10.1107/S2056989023009805 PMC10833403 38313129

[bb7] Roodt, A. (2024). *Acta Cryst.* E**80**, 94–101. 10.1107/S2056989023010757 PMC10848975 38333125

[bb8] Scandolo, S., Mtingwa, S. & Zema, M. (2017). *Acta Cryst.* A**73**, C1069.

[bb9] Zema, M., Desiraju, G. R., Lecomte, C., Nalecz, M. & Ngome Abiaga, J. J.-P. (2017). *Acta Cryst.* A**73**, C363.

[bb10] Zema, M. & Lecomte, C. (2015). *Acta Cryst.* A**71**, s177.

